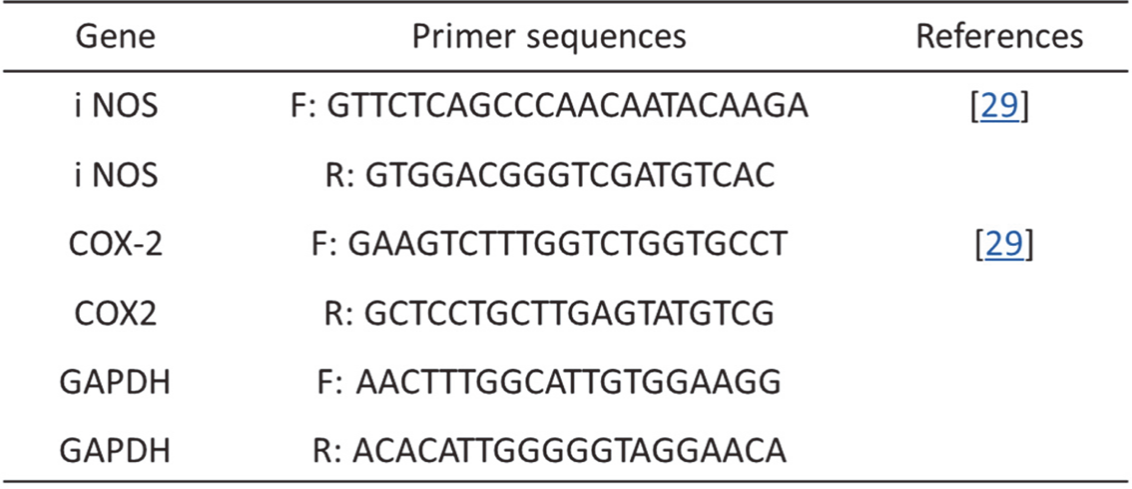# *Latilactobacillus curvatus* BYB3 Isolated from Kimchi Alleviates Dextran Sulfate Sodium (DSS)-Induced Colitis in Mice by Inhibiting IL-6 and TNF-R1 Production

**DOI:** 10.4014/jmb.2109.09054

**Published:** 2021-12-29

**Authors:** Xing Wang, Dingyun Li, Ziyao Meng, Kiyeop Kim, Sejong Oh

**Affiliations:** Division of Animal Science, Chonnam National University, Gwangju 61186, Republic of Korea

**Keywords:** *Latilactobacillus curvatus*, immune modulation, IL-6, probiotic

## Abstract

Recent studies have shown that probiotics have health-promoting effects, particularly intestinal immune modulation. In this study, we focused on the immunomodulatory properties of *Latilactobacillus curvatus* BYB3, formerly called *Lactobacillus curvatus*, isolated from kimchi. In a mouse model of 14-day dextran sulfate sodium (DSS)-induced colitis, treatment with *L. curvatus* BYB3 significantly decreased the disease activity index, colon length, and weight loss. Moreover, histological analyses showed that *L. curvatus* BYB3 protected the structural integrity of the intestinal epithelial layer and mucin-secreting goblet cells from DSS-induced damage, with only slight infiltration by immune cells. To evaluate the molecular mechanisms underlying *L. curvatus* BYB3&ndash;driven inhibition of interleukin 6 production, possible in vivo anti-inflammatory effects of *L. curvatus* BYB3 were examined in the same mouse model. In addition, significantly lower levels of IL-6 and tumor necrosis factor receptor 1 upregulation were seen in the DSS+BYB3 group (compared to that in the DSS group). These results indicate that *L. curvatus* BYB3 exhibits health-promoting effects via immune modulation; and therefore, it can be used to treat various inflammatory diseases.

## Introduction

Inflammatory bowel disease (IBD) refers to a group of inflammatory conditions affecting the colon and small intestine, *e.g.*, Crohn’s disease and ulcerative colitis [1]. There has been an increase in the incidence and prevalence of IBD in Asia since the early 1990s [2]. A family history and several environmental variables are risk factors for IBD, with the susceptible population being young adults [3]. Patients with IBD experience clinical gastrointestinal symptoms and a chronic disease–associated emotional burden characterized by reduced quality of life and ability to work; IBD is responsible for economic losses as well [4]. Although immune factors affect the pathogenesis of IBD, few reports have addressed the possible involvement of interleukin 6 (IL-6) and tumor necrosis factor receptor 1 (TNF-R1).

IL-6 is a pleiotropic cytokine produced by monocytes, macrophages, and activated T cells, B cells, and other cells [5]. Cytokines such as IL-6 and cytokine receptors such as TNF-R1 play key roles in the regulation of the innate immune response after bacterial meningitis [6] and have also been implicated in the pathogenesis of IBD [7]. IBD is associated with the proinflammatory cytokines IL-6 and TNF-α as well as with proinflammatory enzymes that induce inflammation, *e.g.*, iNOS and COX-2 [8]. Several murine models of colitis have been developed to study the mechanism underlying human IBD. Among the various models of chemically induced colitis, the dextran sodium sulfate (DSS)-induced colitis model is widely used because of its simplicity and numerous similarities with human ulcerative colitis [9].

Gut microbes play a very important role in gut health. Probiotics consist of individual or multiple live bacterial species, such as lactobacilli and bifidobacteria, that directly alter the gut microbiota [10, 11]. Numerous studies have revealed that *Lactobacillus* species ameliorate colitis by influencing the host immune responses, mainly via inhibition of the overexpression of inflammatory factors [12-14]. In the present study, *Latilactobacillus curvatus* BYB3 (isolated from kimchi) significantly decreased the IL-6 and TNF-R1 levels and the disease activity index (DAI) during DSS-induced colitis in mice. The DSS+BYB3 group mice recovered almost to the same level as the control group mice (no treatment and no colitis).

## Materials and Methods

### Animals

**Fig. 1:**
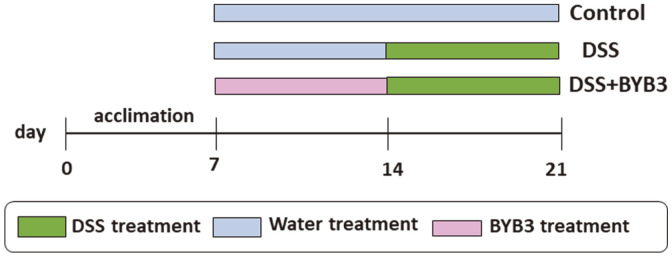
An overview of the experimental design. The experimental design for evaluating the therapeutic effects of *Latilactobacillus curvatus* BYB3 on dextran sulfate sodium (DSS)-induced colitis in a mouse model. Eighteen mice were divided into three groups (*n* = 6 each), *i.e.*, the control, DSS, and DSS+BYB3. DSS+BYB3 mice were fed the normal chow diet and drinking water containing 4% DSS and were orally administered 10^9^ colony-forming units (CFU)/ml *L. curvatus* BYB3 (prepared in 200 μl phosphate-buffered saline (PBS)) for 1 week, *i.e.*, from day 14 to day 21, whereas the DSS group mice were fed the normal chow diet and drinking water containing 4% DSS for 1 week, *i.e.*, from day 14 to day 21, with the simultaneous oral administration of 200 μl of PBS. The control mice were fed the normal chow diet and drinking water and were provided PBS (200 ml) by oral gavage throughout the experiment.

C57BL/6J mice (5 weeks old, female) were purchased from Orient Bio (Korea). The mice were maintained under the following conditions: temperature, 22‒25°C; humidity, 50–60%; 12 h light/dark cycle. The specific experimental design is illustrated in Fig. 1. The mice were provided with sufficient water and a standard chow diet and acclimatized to the laboratory conditions for 7 days before experimentation. They were subdivided into control, DSS, and DSS+BYB3 groups, with six mice in each group (*n* = 18). The control group received a normal chow diet. The DSS group received a normal chow diet and drinking water containing 4% DSS for 1 week (from day 14 to day 21), with simultaneous oral administration of DSS in 200 μl Phosphate-buffered saline (PBS) (to match the group described next). The DSS+BYB3 group received a normal chow diet and drinking water containing 4% DSS for 7 days (as above), and *L. curvatus* BYB3 (10^9^ colony-forming units [CFU]/ml prepared in 200 μl of PBS) was administered orally (once daily) from day 7 to 21. The DAI was assessed daily based on the following formula (Table 1): DAI = (Body weight loss) + (Diarrheal score) + (Rectal bleeding score) [15]. On day 21, all mice were euthanized. The length of the colon was measured, and the colon contents were carefully collected. Clinical features, including watery diarrhea—often with blood—abdominal discomfort, fever, and anemia were scored separately, and correlations with the histological score were examined. The animal experiments were approved by the Institutional Animal Care and Use Committee of Chonnam National University (CNU IACUC-YB-2021-76).

### Reagents

DSS (molecular weight, 36–50 kDa) was procured from MP Biomedicals (Canada). Alcian blue (lot # MKCH7193, Sigma, USA), eosin Y (lot # 6G1490, Junsei Chemical, Japan), phloxine B (lot # 5H1471, Junsei, Japan), and Nuclear Fast Red (Kernechtrot; C.I. 60760, Croatia) were also used in this study.

### Bacterial Identification Using 16S Ribosomal RNA (rRNA) Analysis

Genomic DNA of *L. curvatus* BYB3 was extracted using the PureHelix Genomic DNA Prep Kit (Nanohelix, Korea). The almost complete 16S ribosomal DNA (rDNA) region of the selected strain was analyzed by Solgent (Korea) by using the following primers: 27-Forward (5′-AGAGTTGATCMTGGCTCAG-3′) and 1492-Reverse (5′-TACGGYTACCTTGTTACGACTT-3′) for polymerase chain reaction (PCR) amplification and sequencing as detailed on the Solgent service website (http://info.solgent.com/guest/ko/16s_order_list_arr.php?Xi=8dk26u14k14u74s&posAreaStart=0&pagePerItem=10&sDate=20210502&eDate=20211102).

### Preparation of the Bacterial Strain

*L. curvatus* BYB3 was isolated from hand-made kimchi in Gwangju (Korea). One day before the experiment, 100 μl of inoculum from the stock solution was added to 10 ml of fresh de Man, Rogosa and Sharpe broth (MRS; Difco, USA). Microbial suspensions were incubated for 24 h at 37°C in sterile closed test tubes to obtain microaerophilic conditions. Activated strains were passaged two or three times and then centrifuged at 1,500 ×*g* for 15 min at 23°C. After the supernatant was discarded, probiotic cells were resuspended in 200 μl of PBS.

### Histological Assessment

The collected colons were washed several times with sterile PBS until the stool was cleared. A part of the colon tissue was then fixed with 10% phosphate-buffered formalin overnight. After fixation, the tissue samples were dehydrated using a low-to-high‒concentration ethanol series. Then, the tissue was paraffin embedded. The paraffin blocks were sectioned (5 μm thick) and stained with hematoxylin–eosin for histological assessment. For mucosal-layer evaluation, Alcian blue was used to stain mucin in the paraffin-embedded sections, and nuclei were counterstained with Nuclear Fast Red.

### ELISA

Whole mouse blood was collected in tubes containing heparin lithium and placed on ice. The blood samples were allowed to clot for 2 h at room temperature before centrifuging for 20 min at 2,000 ×*g*. As a result, whole blood separated into layers, and the upper layer of hyaluronic plasma was collected for the next experiment. Then, supernatant fractions were collected for determining the levels of IL-6, TNF-R1, TNF-R2, and TNF-α using respective ELISA kits (R&D Systems, USA) in accordance with the manufacturer’s instructions.

### RNA Isolation and Gene Expression Analysis

Total RNA was extracted from the excised mouse colon using the RNeasy Mini Kit (Qiagen, USA) according to manufacturer’s protocol. Next, 2 μg of total RNA was reverse transcribed into complementary DNA (cDNA) using the Maxime RT Premix Oligo (dT) RT-PCR Kit (iNtRON Biotechnology, Inc., Korea). The primers used in the study are listed in Table 2. Glyceraldehyde-3-phosphate dehydrogenase (GAPDH) served as the internal control. Quantitative PCR was performed on a thermal cycler (Bio-Rad Laboratories, USA). The PCR conditions were as follows: 95°C for 5 min, one cycle; followed by 40 cycles of 95°C for 5 s, 60°C for 30 s, and 72°C for 1 min. Relative gene expression levels were determined by comparative analysis using the formula:

Relative expression = 2^-(ΔΔCt)^, where *C_t_* = *C_t gene_* – *C_t GAPDH_*.

### Statistical Analyses

All data are presented as the mean ± SD from triplicates. Statistical significance of the differences between groups of mice was determined using Student’s *t*-test. In experiments comparing multiple groups, the significance of differences among groups was evaluated using one-way analysis of variance. Statistical analyses were performed using SPSS version 20 (SPSS, Inc., USA), and a *p*-value of <0.05 was considered significant.

## Results

### 16S rDNA Identification

Analysis of 16S rDNA sequences using BLAST showed that the BYB3 strain shares 96% homology with *L. curvatus*. Further, a close grouping of BYB3 with the other eight *L. curvatus* strains was observed, in agreement with the BLAST results. Thus, our strain’s identity as *L. curvatus* BYB3 was validated successfully.

### *L. curvatus* BYB3 Ameliorates the Disruption of Intestinal Barrier Function in the Murine Model of DSS-Induced Colitis

**Fig. 2:**
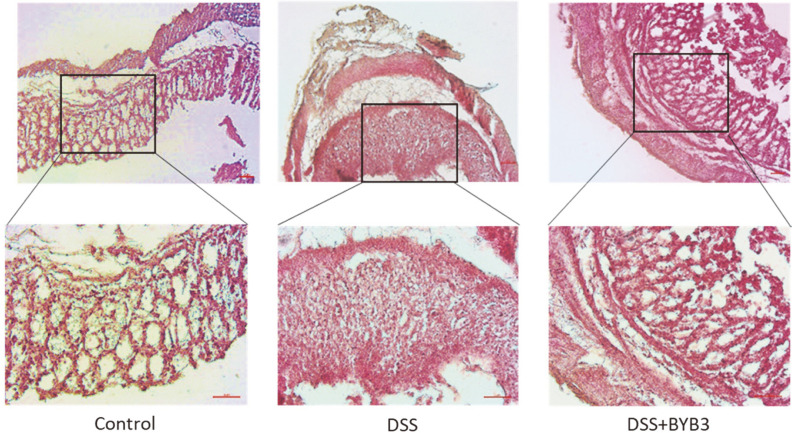
Hematoxylin–eosin staining of the colon sections from mice with DSS-induced colitis. Colons from the control, DSS-treated, and DSS+*L. curvatus* BYB3–treated groups were stained with hematoxylin–eosin. A well-pronounced loss of intact epithelial structure and strong infiltration of immune cells were observed in the DSS group. The DSS+BYB3 group exhibited only slight epithelial structural damage and immune-cell infiltration. Scale bar = 50 μm for 10× and 20× magnification.

To determine whether *L. curvatus* BYB3 ameliorated DSS-induced colitis in mice, colon tissues were histologically examined. In the control group, the mucosal tissue was free of inflammatory infiltration. By contrast, the DSS-treated intestinal tissue showed inflammatory infiltration and damage to the epidermal structure. These aspects were greatly ameliorated in mice treated with both DSS and *L. curvatus* BYB3, *i.e.*, only slight epithelial structural damage and immune-cell infiltration could be observed (Fig. 2).

**Fig. 3:**
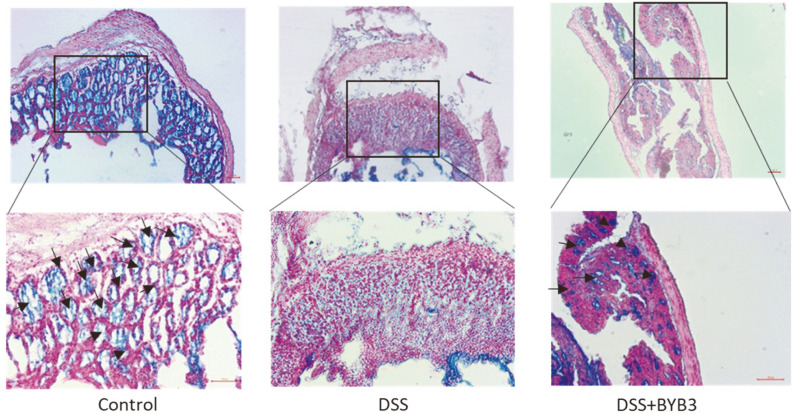
Alcian blue staining of colon sections from mice with DSS-induced colitis. Mucin-secreting goblet cells (arrowhead) were found to be abundantly distributed in the colon tissue of the control group. In the DSS group, the number of mucin-secreting goblet cells was much smaller, with a limited number of goblet cells (arrowhead) scattered around the colon. *L. curvatus* BYB3 protected the colon against the DSS-induced damage by increasing the number of mucin-secreting goblet cells in the colon. Scale bar = 50 μm for 10× and 20× magnification.

The micrographs of Alcian blue–stained colon sections (Fig. 3) revealed that the mucous layer and goblet cells were normal and well preserved in the control group. The mucous layer was found to be disrupted in the DSS group. By contrast, a large number of goblet cells and only a slight alteration of mucus integrity were observed in the DSS+BYB3 group.

### *L. curvatus* BYB3 Alleviates Symptoms of DSS-Induced Colitis

**Fig. 4:**
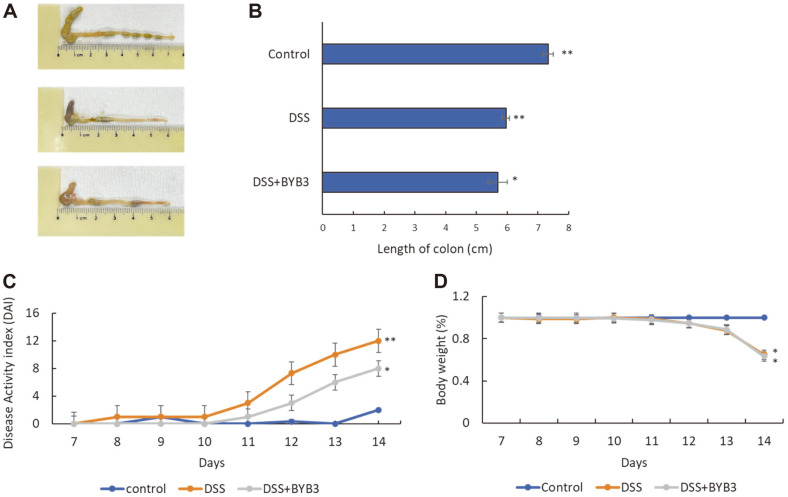
*Latilactobacillus curvatus* BYB3 alleviates symptoms of DSS-induced colitis. (**A, B**) Images of the colons dissected from the control, DSS, and DSS+BYB3 groups. (**C**) The disease activity indices (DAIs) of the mouse colons were calculated based on body weight loss, stool consistency, and abnormal or gross blood-related characteristics. (**D**) Body weight changes were monitored and are depicted as a percentage of the initial body weight. The values represent the mean ± SEM from six mice. The results are representative of three separate experiments. Significant differences from the control group, *p < 0.05, **p < 0.01.

Alleviation of symptoms was assessed by investigating the epidermal structure and inflammatory infiltration of the colon tissue while considering colon length. The control group exhibited the highest colon length (7.3 ± 0.3 cm), the cecum was more complete than that in the other two groups, and the stool appeared healthier. The DSS group had shorter average colon length (5.9 ± 0.2 cm) with a dark red color and intestinal hemorrhage. The colons of the mice in the DSS+BYB3 group had less-than-normal average length, *i.e.*, 5.8 ± 0.4 cm. There was no significant difference in the colon length between the DSS group and the DSS+BYB3 group. Nonetheless, the colons from the DSS+BYB3 group had a lighter color than those from the DSS group. Moreover, the colon of the colitis-affected mice treated with BYB3 exhibited significantly attenuated intestinal bleeding as compared to that in the DSS group (Figs. 4A and 4B). DAI combined scores for weight loss, stool consistency, and bleeding showed that disease activity was markedly lower in the DSS+BYB3 group (Fig. 4C). Importantly, weight loss was not attenuated in these mice (Fig. 4D). These findings indicated that strain BYB3 ameliorated inflammation in DSS-induced colitis.

### *L. curvatus* BYB3 Alleviates DSS-Induced Colitis in Mice by Inhibiting the Production of IL-6, TNF-R1, TNF-R2, and TNF-α

**Fig. 5:**
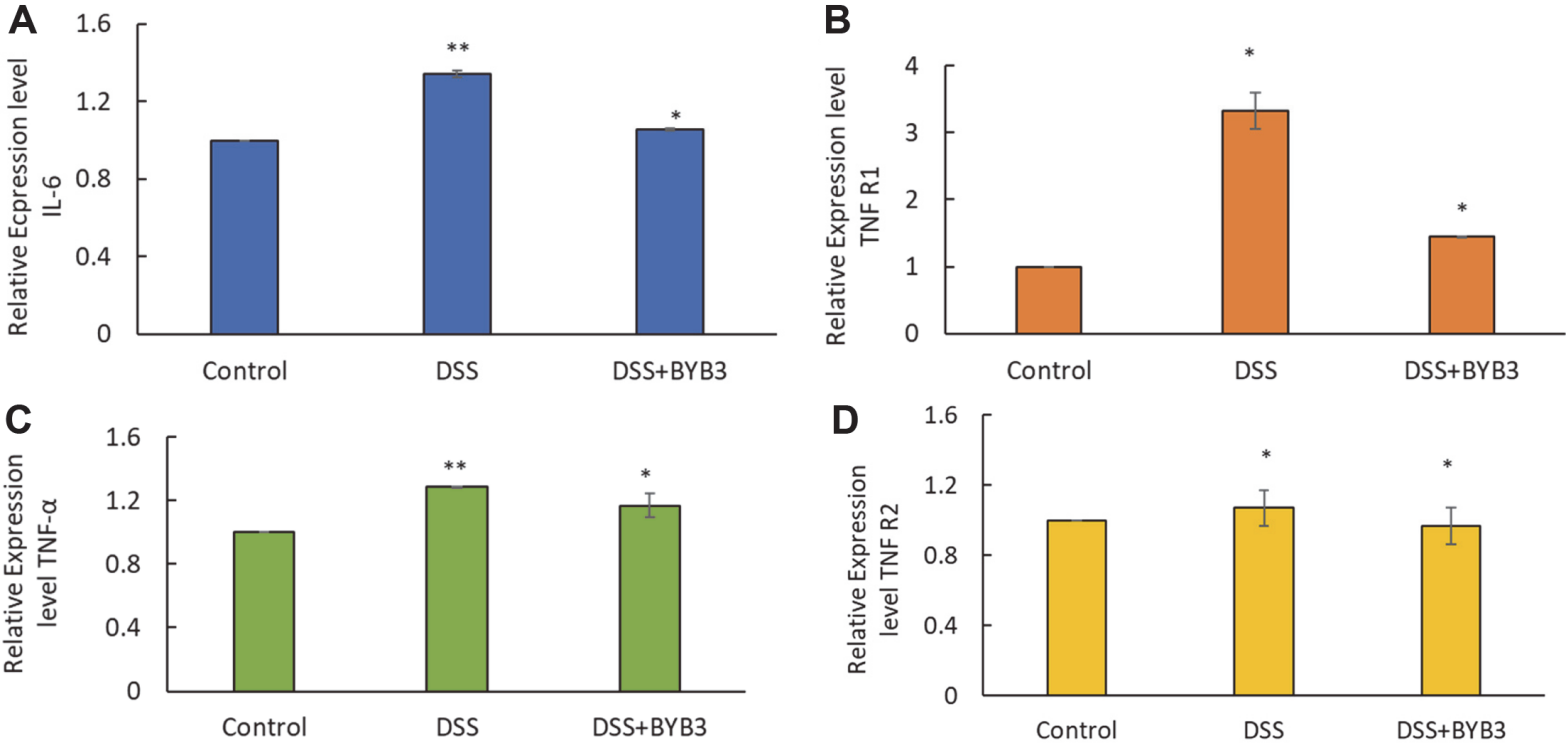
Relative expression levels of inflammatory cytokines. Relative expression of inflammatory cytokines (IL6 and TNF-α) and two types of TNF-a receptors (TNF-R1 and TNF-R2) in blood serum of mice with DSS-induced colitis. The values represent the mean ± SEM from six mice. Significant differences from the control group, *p < 0.05, **p < 0.01.

As shown in Fig. 5, IL-6, TNF-R1, TNF-R2, and TNF-α levels were significantly increased in response to DSS treatment. The upregulation of proinflammatory cytokines proved to be significantly attenuated in the DSS+BYB3 group. Moreover, the relative TNF-R1 expression level was found to be significantly reduced in the DSS+BYB3 group (compared with the DSS group) but remained higher than that in the control group. Furthermore, IL-6 overexpression was significantly attenuated in the DSS+BYB3 group compared to that in the DSS group, but the IL-6 level remained higher in the DSS+BYB3 group than in the control group. TNF-R2 and TNF-α expression levels increased less than the TNF-R1 expression level in the DSS group; however, in the DSS+BYB3 group, they returned to a level similar to that observed in the control group.

### *L. curvatus* BYB3 Decreases the Expression of iNOS and COX-2 mRNA in the Colon Tissue

**Fig. 6:**
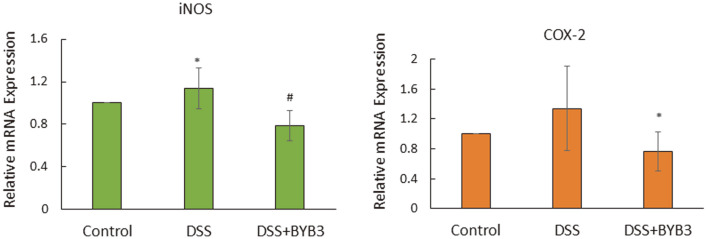
Relative mRNA expression levels of iNOS and COX-2 in test groups (DSS and DSS+BYB3) . Relative transcript-level expression of proinflammatory enzymes (iNOS and COX-2) in the colon of mice with DSS-induced colitis. The values represent the mean ± SEM from six mice. The results are representative of three separate experiments. Significant differences from the control group, *p < 0.05, **p < 0.01.

Compared with that in the control group, the expression of iNOS, and COX-2 mRNA was significantly higher in the DSS group. The mRNA overexpression of iNOS and COX-2 was significantly attenuated in the DSS+BYB3 group. Similar to the expression of COX-2 mRNA, the expression of iNOS mRNA was also lower in the DSS+BYB3 group compared to that in the DSS group, but the attenuation of iNOS expression was not evident, as shown in Fig. 6.

## Discussion

There are many types of intestinal microbes; the growing recognition of the importance of the role played by beneficial bacteria and research on the inhibition of harmful bacteria are particularly important with respect to human health. Several studies indicate that probiotics can alleviate colitis; for example, *L. curvatus*, *L. acidophilus*, *L. casei*, *L. brevis*, and *L. fermentum* can directly promote recovery from colitis [16, 17]. *L. curvatus* has also exhibited robust performance in other applications, *i.e.*, it can be used in fermented foods such as sausages [18]. Recently, it was demonstrated that *L. curvatus* strains have a high freeze tolerance [19], and this feature makes them highly suitable for low-temperature storage. With advancements in research on the characteristics of probiotics, we were interested in evaluating their application in mice.

Inflammation is a protective response of the host immune system. However, uncontrolled inflammatory responses can result in host tissue injuries. The DSS-induced inflammatory response in murine macrophages is an in vivo model that is extensively used for studying anti-inflammatory factors [20, 21]. In this study, DSS was used to induce colitis in mice. The clinical symptoms of DSS-induced colitis in mice are similar to those observed in patients with IBD, and include smaller colon length, colon pathology, and elevated inflammatory biomarker levels [22]. Recent research suggests that probiotics can prevent IBD in both experimental models and humans. Studies have shown that orally administered *L. curvatus* increases the survival rate of mice with DSS-induced colitis and reduces the severity of the clinical symptoms and histopathological changes in colon tissues [23]. Our current results revealed that *L. curvatus* BYB3 ameliorates DSS-induced loss of body weight and inflammatory damage to the colon in mice, which is in agreement with our current result that the DAI score was significantly lower in the DSS+BYB3 group. Weight loss, bloody diarrhea, shortened colon length, colonic bleeding, and destruction of intestinal epithelial structure are commonly observed in mice with DSS-induced colitis [24]. In this study, *L. curvatus* BYB3 reduced colon bleeding while restoring the normal intestinal epithelial structure, but had no effect on the reduced colon length. Histological analysis showed that *L. curvatus* BYB3 reduced colon bleeding in the mice with DSS-induced colitis by maintaining the integrity of epithelial structure and by preventing substantial immune-cell infiltration. Damage to goblet cells in the colon resulted in a lower DAI score in the DSS+BYB3 group than in the control group.

In acute DSS-induced colitis, the immunological pathogenesis involves abnormal and uncontrolled upregulation of inflammatory cytokines. In other reports, both TNF-a and IL-6 have been investigated as the main molecules of interest. As a proinflammatory cytokine, IL-6 modulates anti-inflammatory mechanisms by targeting NF-κB, thereby inhibiting the cellular production of TNF-α, which could be one of the mechanisms underlying NF-κB suppression that we have observed in an anti-inflammatory state [25]. In our current study, *L. curvatus* BYB3 significantly weakened the DSS-induced overexpression of colonic TNF-R1 and IL-6; this effect was likely mediated by the attenuation of activated monocyte and macrophage infiltration (primary sources of these two proteins) [26]. A few reports have indicated the importance of IL-6 in the pathophysiology of experimental colitis and human IBD [27, 28]. This finding corroborates our results. COX-2 is induced by a variety of harmful factors and is involved in mediating the inflammatory response by catalyzing the synthesis of certain prostaglandins. A variety of stimulatory factors increase the induced expression of iNOS, thereby leading to the synthesis of large amounts of NO and promoting inflammation. Many authors have confirmed that COX-2 and iNOS are related to the pathogenesis of ulcerative colitis [29, 30].

In conclusion, *L. curvatus* BYB3 isolated from the fermented food kimchi exerts considerable beneficial effects and relieves colitis in mice by inhibiting the expression of a proinflammatory cytokine and receptor that disrupt intestinal epithelial structural integrity. Our findings suggest that *L. curvatus* BYB3 ameliorates colitis by reducing the inflammation caused by the overexpression of a proinflammatory cytokine. Further investigation is needed to clarify the role of inflammatory cytokines in IBD pathogenesis.

## Figures and Tables

**Table 1:** The scoring system used for calculating the disease activity index (DAI).

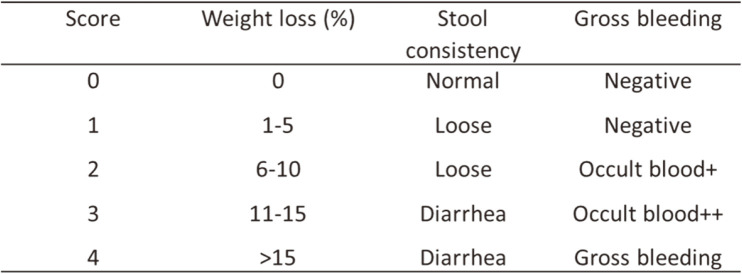

**Table 2:** Primer sequences for quantitative polymerase chain reaction (PCR).